# Genome-wide identification and expression analysis of the NAC transcription factor family in tomato (*Solanum lycopersicum*) during aluminum stress

**DOI:** 10.1186/s12864-020-6689-7

**Published:** 2020-04-07

**Authors:** Jian Feng Jin, Zhan Qi Wang, Qi Yu He, Jia Yi Wang, Peng Fei Li, Ji Ming Xu, Shao Jian Zheng, Wei Fan, Jian Li Yang

**Affiliations:** 1 State Key Laboratory of Plant Physiology and Biochemistry, Institute of Plant Biology, College of Life Sciences, Zhejiang University, Hangzhou, 310058 China; 2 Key Laboratory of Vector Biology and Pathogen Control of Zhejiang Province, College of Life Sciences, Huzhou University, Huzhou, 313000 China; 3 College of Resources and Environment, Yunnan Agricultural University, Kunming, 650201 China

**Keywords:** Tomato, NAC family, Phylogenetics, Expression profile, Al stress, Stress response

## Abstract

**Background:**

The family of NAC proteins (NAM, ATAF1/2, and CUC2) represent a class of large plant-specific transcription factors. However, identification and functional surveys of *NAC* genes of tomato (*Solanum lycopersicum*) remain unstudied, despite the tomato genome being decoded for several years. This study aims to identify the *NAC* gene family and investigate their potential roles in responding to Al stress.

**Results:**

Ninety-three *NAC* genes were identified and named in accordance with their chromosome location. Phylogenetic analysis found *SlNACs* are broadly distributed in 5 groups. Gene expression analysis showed that *SlNACs* had different expression levels in various tissues and at different fruit development stages. Cycloheximide treatment and qRT-PCR analysis indicated that *SlNACs* may aid regulation of tomato in response to Al stress, 19 of which were significantly up- or down-regulated in roots of tomato following Al stress.

**Conclusion:**

This work establishes a knowledge base for further studies on biological functions of *SlNACs* in tomato and will aid in improving agricultural traits of tomato in the future.

## Background

Aluminum (Al) is the most abundant metal element in the earth’s crust. Although it is nontoxic when it exists in oxides or hydroxides in neutral and alkaline conditions, the solubility of Al increases dramatically when soil pH is lower than 5.5, and solubilized Al is highly toxic to most plant species [1]. However, nearly 30% of arable lands and 50% of potentially arable lands are estimated to be acidic [2]. Therefore, Al toxicity is well recognized as one of the major edaphic factors threatening food security worldwide [1]. To survive the acidic Al toxic environment, plants have developed complicated coping mechanisms, which are largely controlled by transcriptional regulation in response to Al stress [3].

Al-induced changes in gene expression occur within hours of exposure in the root apex of some plant species, suggesting that transcriptional regulation is vital for plants to adapt to the stress [4–6]. Plant transcription factors (TFs) are central regulators that direct transcription via binding to special nucleotide sequences in response to developmental cues and environmental stresses [7]. Since the first report on an *Arabidopsis* mutant hypersensitive to both low pH and Al, STOP1 (Sensitive to proton rhizotoxicity 1) and its homologous genes from other plant species have been well-documented as a very important TF regulating several critical processes involved in Al tolerance [8]. In addition, several other TFs have also been characterized and implicated in Al tolerance. However, the majority are demonstrated to play minor roles in regulation of the expression of genes involved in organic acid anion secretion [8]. For example, whilst *AtALMT1* (Al-activated malate transporter 1) expression was predominantly controlled by STOP1, CAMTA2 (CALMODULIN-BINDING TRANSCRIPTION ACTIVATOR2) and WRKY46 had a positive and negative role, respectively, in regulating *AtALMT1* expression under Al stress [9, 10]. Although ART1 (Al resistance transcription factor 1) is a master TF controlling the expression of Al-tolerance genes including *OsFRDL4* in rice, WRKY22 was recently reported to bind to the promoter of *OsFRDL4* and regulate its expression [11]. However, other TFs in Al tolerance remain to be characterized.

As an important class of TFs, NAC, which is a descendent of 3 proteins of NAM (No apical meristem), ATAF 1/2 (Arabidopsis transcription activator factor 1/2) and CUC2 (Cup shaped cotyledon) [12], is a class of plant specific TFs and constitute one of the largest TF families in plants [13]. Typically, NAC TFs have a conserved NAM domain at the N-terminus and a diverse transcription regulatory region at the C-terminus [14]. It has been shown that NAC TFs have a crucial position not only in plant development and growth, but also in stress responses [15, 16].

Recently, several lines of evidence suggest the implication of NAC TFs in response to Al stress in plants. For instance, 25 *NAC* genes were found to be differentially expressed among different rice genotypes in response to Al stress and most of these *NAC* genes belong to the NAM subfamily [17]. We previously identified a *NAC* transcription factor gene up-regulated by Al stress in the root apex of rice bean [4]. Further functional characterization of this rice bean *NAC* gene showed that it could regulate *WAK1* (Wall-associated protein kinases) expression and cell wall pectin metabolism when ectopically overexpressed in Arabidopsis [18]. SOG1 (SUPPRESSOR OF GAMMA RESPONSE1) is a NAC protein that acts as a central DNA damage response component [19]. Interestingly, SOG1 loss-of-function mutant displayed better root growth in comparison with wild-type plants during long-term exposure to low dosage Al [19]. However, *sog1* mutant became extremely sensitive to Al when higher Al concentrations were applied in the growth medium [20]. Although these results suggest a complexity of responses of Arabidopsis plants to Al-induced DNA damage, it provided solid evidence that a NAC protein, SOG1, is involved in the Arabidopsis response to Al stress.

Tomato (*Solanum lycopersicum*) ranks fourth among the leading world vegetables in production. It is a rich source of nutrients and a model plant for fleshy fruit development [21]. However, with a continuously expanding scale of cultivation of tomato, they have suffered serious damage in recent years, not only caused by abiotic stresses like drought or temperature stress but also various pathogens and pests, such as fungi, insects and nematodes [22]. Unfortunately, few studies have focused on the response of tomato to Al stress. In a previous study, we characterized root organic acid anions secretion from tomato roots [23]; however, the underlying molecular basis is unknown. In the preset study, we aimed to provide a comprehensive view of the *NAC* gene family in tomato and to identify members involved in the response to Al stress.

## Results

### Genome-wide identification and phylogenetic analysis of the *NAC* gene family in tomato

**Fig. 1:**
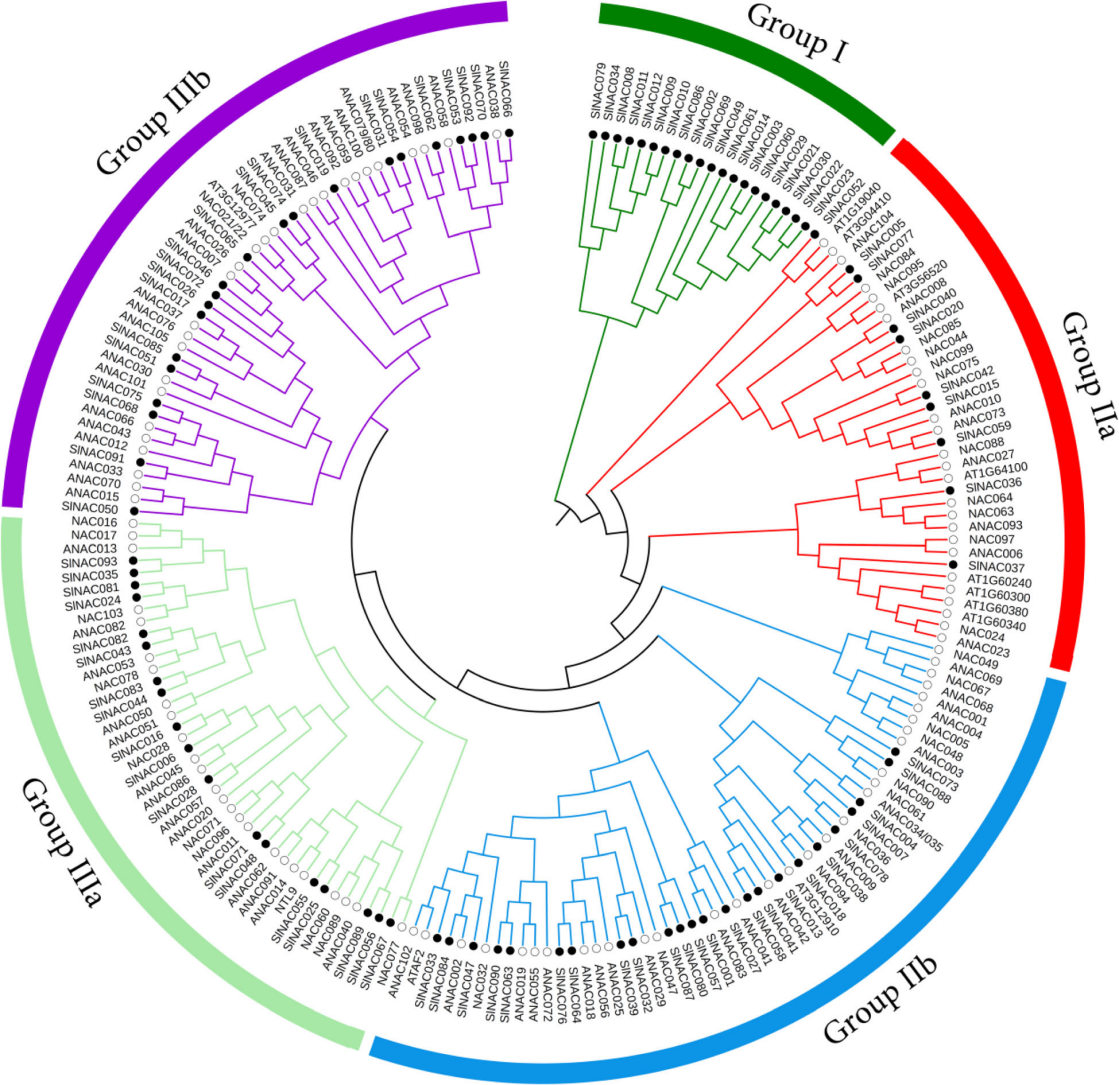
Phylogenetic analysis of tomato (*Solanum lycopersicum*) NACs (SlNACs). Phylogenetic analysis of NACs from tomato and Arabidopsis using the complete protein sequences. The Neighbor-joining (NJ) tree was constructed using MUSCLE and MEGA 7.0 software with the pairwise deletion option and 1000 bootstrap replicates were used to assess tree reliability. NACs from each plant species have colored labels. NACs of different plant species fell in 5 separate subfamilies as Group I, Group IIa, Group IIb, Group IIIa and Group IIIb

In our study, BLAST and HMM searches were performed to broadly identify tomato NAC family using the NAC protein sequences in Arabidopsis and rice as queries. All of the putative proteins fulfilled the criteria of NAC proteins as described in previous research [7, 24]. As a result, 93 putative NAC proteins were identified in the *S. lycopersicum* genome, which were designated as SlNAC1-SlNAC93 based on their locations on the chromosomes (Table S1). The number of amino acid residues of the predicted SlNACs ranged from 108 to 1029, and their molecular mass varied from 12.28 to 117.0 kDa (Table S1). To probe the phylogenetic relationships among these 93 SlNACs, a phylogenetic tree was constructed by combining SlNACs with Arabidopsis NAC proteins (AtNACs). Because sequence lengths varied dramatically, phylogenetic tree was constructed based on maximum likelihood algorithm following [7]. The results indicated that the NAC family could be divided into 5 subfamilies (Group I, Group IIa, Group IIb, Group IIIa, and Group IIIb) (Fig. 1). Group III was the largest with 39 SlNACs and 2 subgroups (IIIa and IIIb) followed by groups II with 34 proteins and 2 subgroups (IIa and IIb) and Group I including 20 NACs was a species-specific subgroups of tomato (Fig. 1). These results suggest that these NACs may have crucial roles in the evolution of the tomato genome.

### Gene structure and protein motif analysis of *SlNAC* genes

**Fig. 2:**
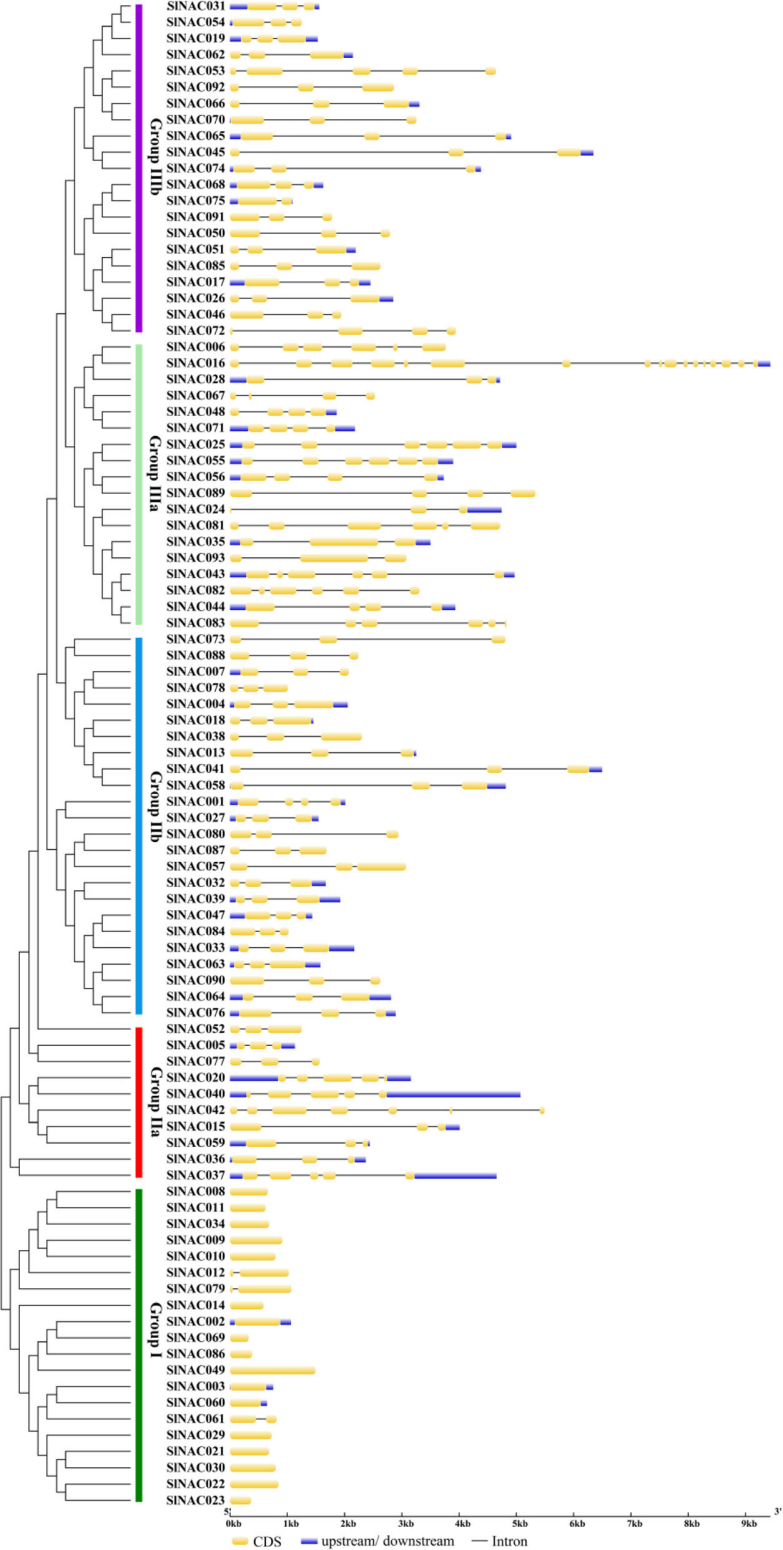
The exon-intron structure of *SlNAC* genes in accordance to the phylogenetic relationship. The unrooted phylogenetic tree was constructed with 1000 bootstrap based on the full length sequences of SlNACs. Exon-intron structure analysis of *SlNAC* genes was performed by using the online tool GSDS. Lengths of exons and introns of each *SlNAC* gene were exhibited proportionally

During the evolution of multigene families, the diversification of gene structure is responsible for evolving gene new function to adapt to the change of the living environments [25, 26]. To understand the structural diversity of *SlNAC* genes, intron/exon organization and conserved motifs were analyzed as described in previous research [13, 14]. Gene structure analysis showed that among these 93 *SlNAC* genes, 14 had no intron, and the others had at least one intron. Most of *SlNAC* members in the same subfamily displayed similar exon-intron structure (Fig. 2). Interestingly, most numbers in group I had only one exon (Fig. 2). This may be because that they are a specific class of *NACs* of tomato.

**Fig. 3:**
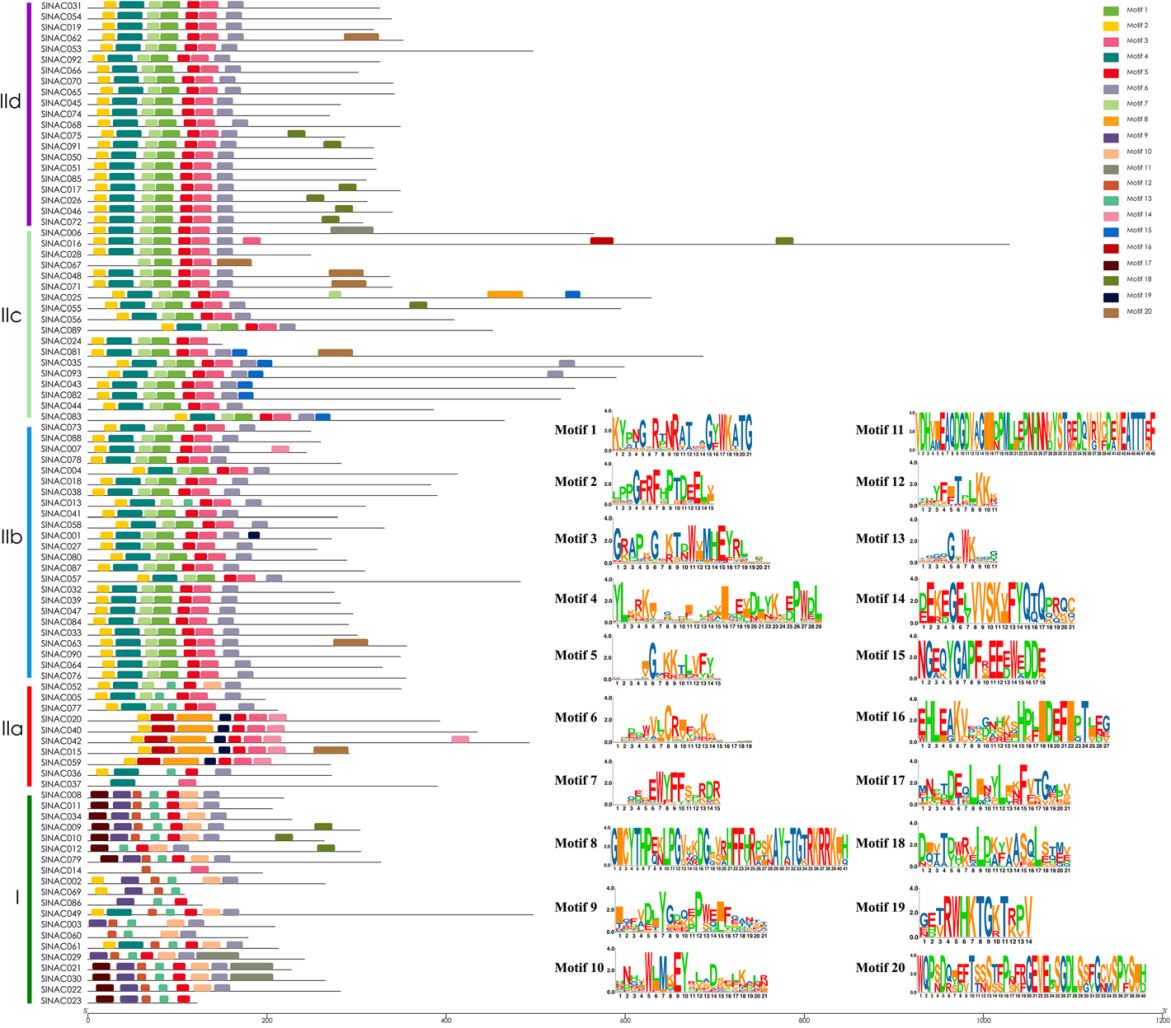
Conserved motifs of SlNAC proteins in accordance to the phylogenetic relationship. The conserved motifs in the SlNAC proteins were identified by MEME. Grey lines represent the non-conserved sequences, and each motif is indicated by a colored box numbered at the bottom. The length of motifs in each protein was displayed proportionally

To further detect potential conserved motifs of SlNAC proteins (SlNACs), we also analyzed the putative motifs using the MEME program as described in previous research [7, 26]. As a result, 20 divergent motifs were identified in SlNACs, which were successively named as motifs 1–20 (Fig. 3). As expected, the closely-related members in the phylogenetic tree generally had mutual motif compositions and only minor differences were observed at subgroup levels (Fig. 3), indicating that there might have functional similarities among the SlNAC proteins within the same subgroup. This is consistent with a previous study showing that *Solanaceae* plants have specific NAC transcription factors [27]. Collectively, these results suggest that SlNACs possessing similar gene structures and motifs were clustered in the same subgroup and might have similar functions in the evolution of tomato.

### Chromosomal distribution and synteny analysis of *SlNAC* genes

**Fig. 4:**
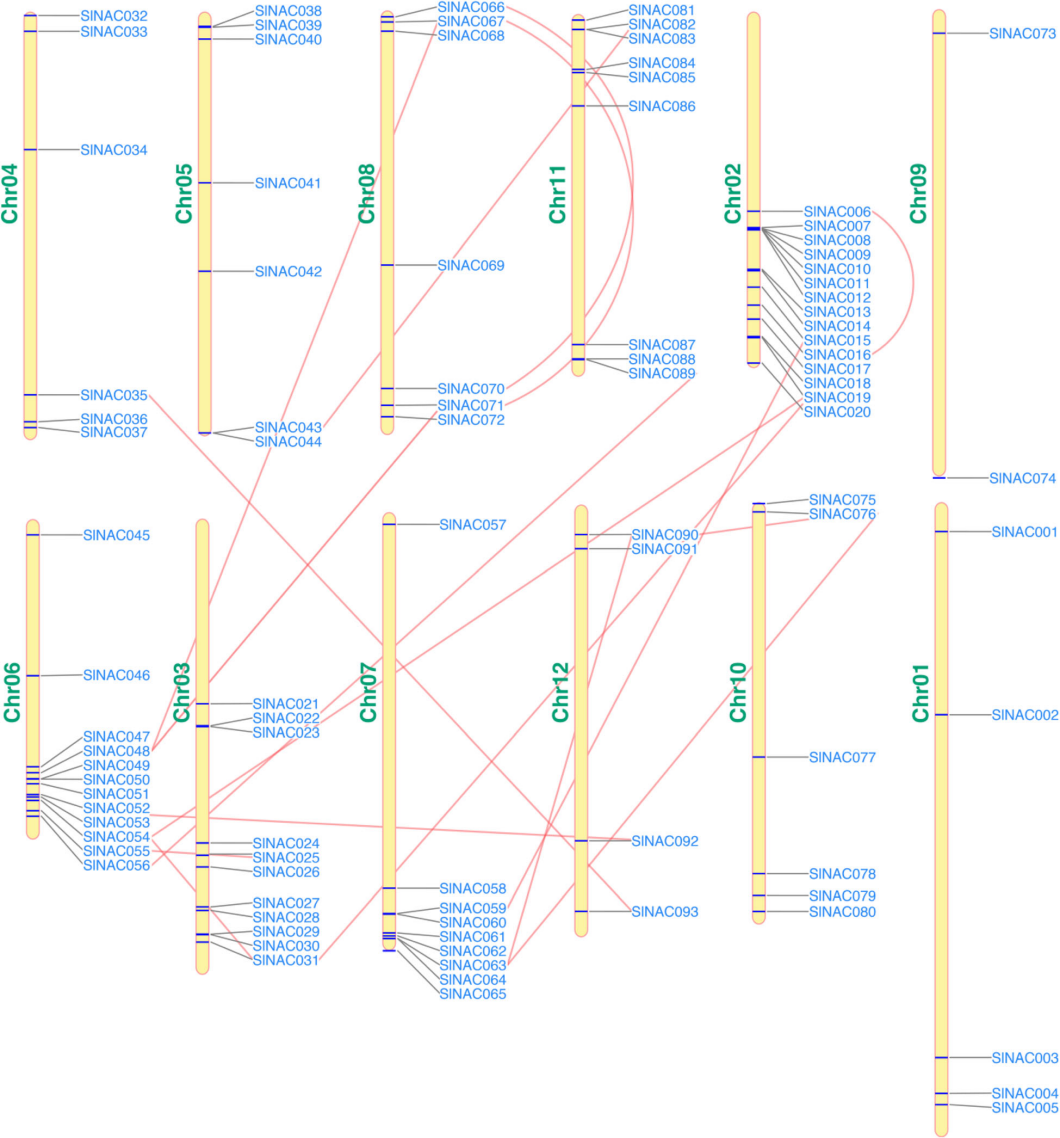
Schematic representations for the distribution and duplication of 93 *SlNAC* genes. Black lines represent the chromosomal location of *SlNAC* genes, and the red lines indicate duplicated *SlNAC* gene pairs. The chromosome number is indicated on the left side of each chromosome

To examine the chromosomal distribution of the *SlNACs*, the genomic sequence of each *SlNAC* was utilized to search against the tomato genome database with BLAST software. Physical map positions demonstrated that all of the 93 *SlNAC* genes could be mapped on 12 chromosomes in increasing order from short arm to long arm telomere (Fig. 4). Although each chromosome encompasses some *SlNAC* genes, the distribution is uneven (Fig. 4). The gene density per Chr (chromosome) ranged from 2.15% (2 *SlNAC* genes on Chr 09) to 16.13% (15 *SlNAC* genes on Chr 02), and relatively low numbers of SlNAC genes were observed in some chromosomes, such Chrs 01 and 12 (Fig. 1).

Furthermore, we also investigated tandem repeats and segmental duplication events of the *SlNAC* genes to explore the mechanism underlying the expansion of the *SlNAC* gene family. In this study, multiple potential pairs linked each of at least 5 tandem repeats and 17 chromosomal segmental duplications were identified (Fig. 4), such as the large sections of Chrs 02 and 07 and Chrs 06 and 08. A previous report has demonstrated that the relatively recent (> 50 million years ago) genome-wide duplication (GWD) has caused a transition of 7 ancestral chromosomes to 12 chromosomes in the tomato [21]. Consistently, we found that there were at least 34 *SlNAC* genes involved in the GWD segment (Fig. 4). These results suggest that some *SlNACs* were possibly produced by gene duplication and the segmental duplication events, which might play a major driving force for *SlNAC* evolution in tomato.

### Tissue specific expression patterns of *SlNACs*

**Fig. 5:**
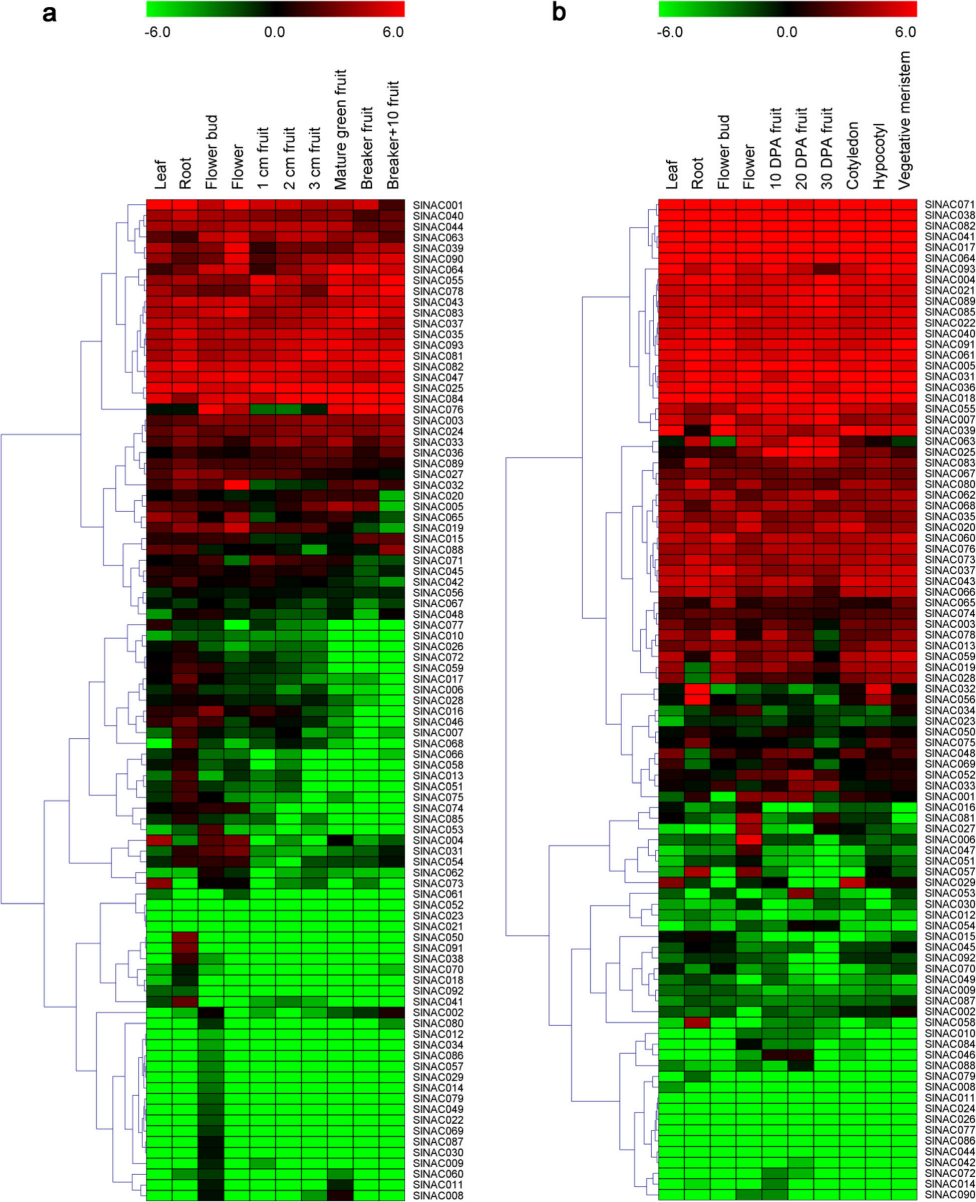
Temporal and tissue-specific expression patterns of 93 *SlNAC* genes. **a** Expression profile of *SlNAC* genes in cultivated tomato cultivar Heniz. **b** Expression profile of *SlNAC* genes in wild species *S. pimpinellifolium*. Expression data were processed with Log2 normalization. The colour scale represents relative expression levels. DPA, days post anthesis

To further explore the expression patterns of the putative *SlNAC* genes, we analyzed their expression profiles in different tissues and development stages of a cultivar Heinz cultivar and wild species *S. pimpinellifolium* using public RNA-seq data [20]. It showed that 96.8% and 94.6.3% of *SlNACs* were expressed in at least one tissue (stage) of Heinz and *S. pimpinellifolium*, respectively (Fig. 5). Twenty-one genes (*SlNAC001*, *SlNAC003*, *SlNAC024*, *SlNAC025*, *SlNAC035*, *SlNAC037*, *SlNAC039*, *SlNAC040*, *SlNAC043*, *SlNAC044*, *SlNAC047*, *SlNAC055*, *SlNAC063*, *SlNAC064*, *SlNAC078*, *SlNAC081*, *SlNAC082*, *SlNAC083*, *SlNAC084*, *SlNAC090*, and *SlNAC093*) were constitutively expressed in all the stages analyzed in the Heinz cultivar, whereas the transcripts of 11 genes (*SlNAC012*, *SlNAC014*, *SlNAC021*, *SlNAC023*, *SlNAC029*, *SlNAC034*, *SlNAC052*, *SlNAC057*, *SlNAC061*, *SlNAC086*, and *SlNAC092*) were hardly detectable. Among these genes, *SlNAC082* had the highest expression level in both the Heinz cultivar and wild species *S. pimpinellifolium* (Fig. 5).

When the expression levels of *SlNACs* in various tested organs were compared between the Heinz cultivar and *S. pimpinellifolium*, 45 showed similar expression patterns in both genotypes of tomato, with 11 genes barely expressed in all tested organs. Conversely, 39 genes showed significant differential expression patterns in the two tomato genotypes (Fig. 5). Notably, the expression of eleven genes was restricted to the leaf (*SlNAC073*) and root (*SlNAC007*, *SlNAC013*, *SlNAC017*, *SlNAC041*, *SlNAC042*, *SlNAC050*, *SlNAC051*, *SlNAC068*, *SlNAC075*, and *SlNAC091*) in Heniz cultivar, whilst only one gene was noted in the root (*SlNAC050*) in *S. pimpinellifolium*. Furthermore, in the Heniz tomato cultivar, expression of three *SlNAC* genes (*SlNAC015*, *SlNAC032*, and *SlNAC076*) was hardly detectable in young tomato fruits (1 cm-, 2 cm-, and 3 cm-fruit), whereas a distinct expression pattern was detected in the breaker fruits (Fig. 5a). In *S. pimpinellifolium*, expression of five *SlNAC* genes (*SlNAC003*, *SlNAC013*, *SlNAC028*, *SlNAC059*, and *SlNAC078*) in young fruits (10 DPA and 20 DPA) was higher than that in breaker fruits (30 DPA) (Fig. 5b). This suggests that the *SlNACs* are regulated in a tissue-specific manner in tomato.

### Expression profiles of *SlNAC* genes in response to Al stress

**Fig. 6:**
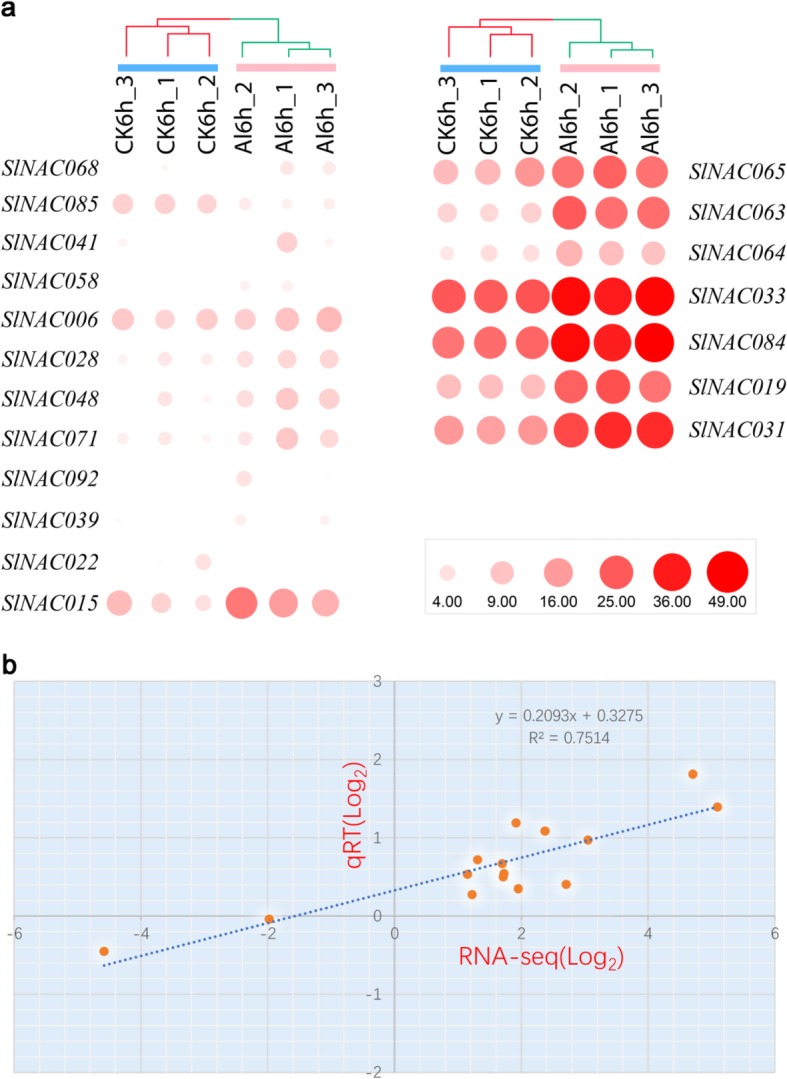
Expression profiles of the *SlNAC* genes under Al stress. **a** Hierachical clustering of expression profiles of *SlNAC* genes during Al stress. **b** Correlation of gene expression levels between RNA-Seq data and qRT-PCR analysis. Fifteen SlNACs potentially responding to Al were selected and subjected to qRT-PCR analysis using the same RNA as for RNA-Seq. Both x- and y-axes are shown in Log2 scale

Following an extensive analysis of *SlNAC* gene family in tomato, we next attempted to investigate the potential implication of *SlNACs* in responding to Al stress. The inhibition of root elongation was the primary visible symptom of Al toxicity and the relative root elongation is widely used to indicate Al toxicity or Al tolerance. Our preliminary experiment indicated that the relative root elongation was about 60% when 5 uM Al was applied for 6 h (Fig. S1), suggesting that 5 uM of Al and 6 h of exposure is suitable for investigating the effects of Al on tomato roots. To this end, the gene expression profiles of *SlNACs* in a tomato cultivar Ailsa Craig were examined using transcriptome analysis. As shown in Table S2, a total of 6 samples were subjected to RNA-Seq and generated about 6.77Gb data for each sample on average. The average genome mapping rate is 87.50% and the average gene mapping rate was 76.22%. Next, clean reads were mapped to the reference genome after merging novel coding transcripts with reference transcripts, and RNA-Seq by Expectation Maximization tool, which was utilized to calculate gene expression levels of both gene and transcript [28]. The number of genes and transcripts of each sample is shown in Table S3. Based on the gene expression level, a total of 1620 up-regulated and 789 down-regulated differentially expressed genes (DEGs) were identified (Fig. S2). The gene lists are shown in Tables S4 and S5 for up- and down-regulated DEGs. Finally, 19 out of 93 *SlNACs* were found to have differential expression patterns after 6-h of exposure to 10 μM Al (Table S6). Among 19 Al-responsive *SlNAC* genes, 7 were found to have relatively high expression levels than others (Fig. 6a). The reliability of the RNA-Seq data was further verified by qRT-PCR analysis which was validated on 15 selected *SlNAC* genes. As shown in Fig. 6b, all of these 15 selected *SlNAC* genes exhibited similar expression patterns to that obtained by RNA-Seq. The Pearson correlation analysis showed a good correlation (*R*^2^ = 0.7514) between RNA-Seq data and qRT-PCR results (Fig. 6b). These results suggest that the RNA-Seq data accurately mirrored the transcriptional changes induced by Al stress.

### Expression of selected *SlNACs* under Al and CHX

**Fig. 7:**
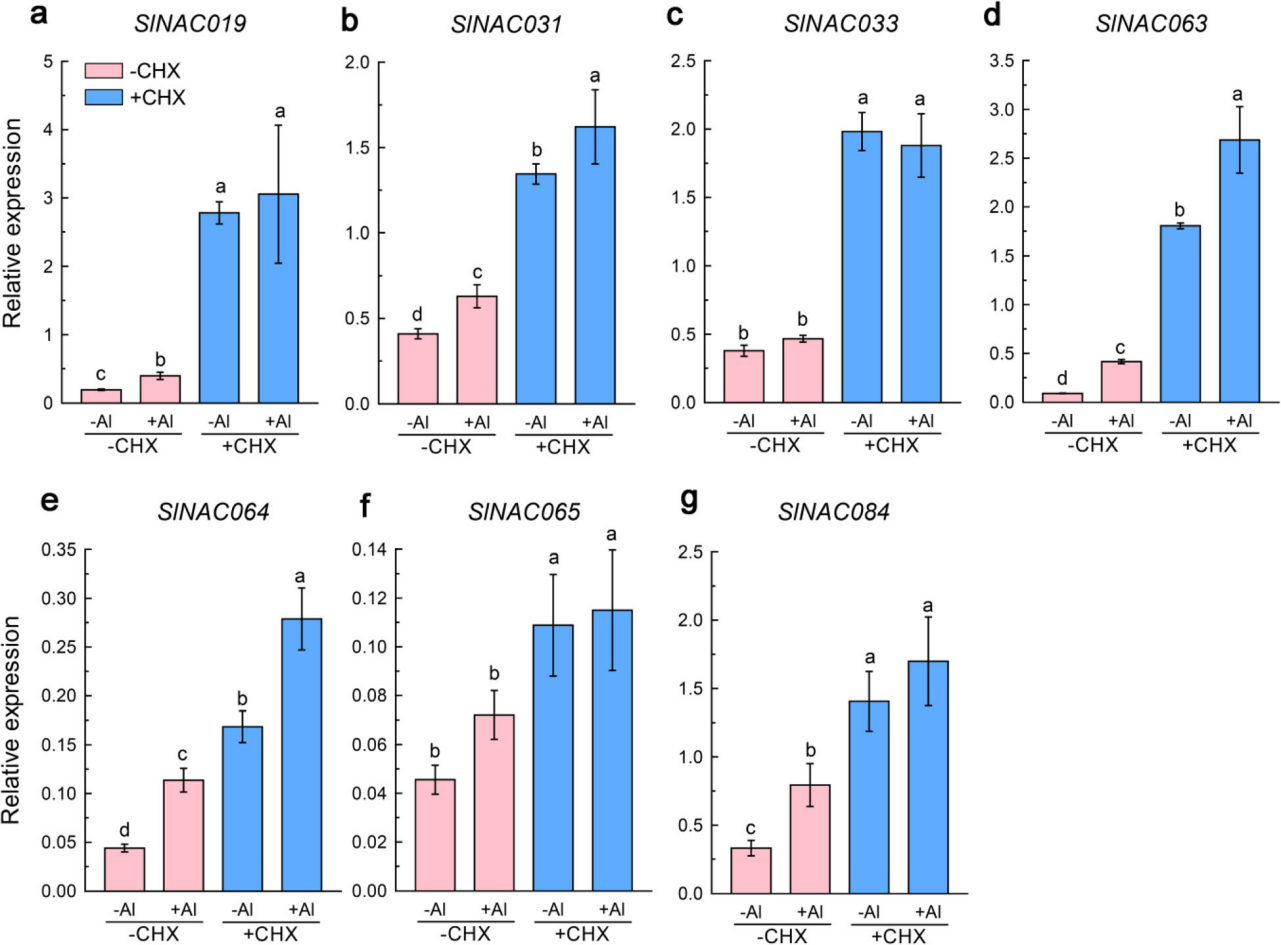
Effects of a protein translation inhibitor, cycloheximide (CHX), on the expression of *SlNAC* genes. Three-day-old seedlings (cv. AC) were subjected to 1/5 strength Hoagland nutrient solution (10 μM Pi; pH 5.0) containing 0 or 5 μM CHX for 1 h, and then the seedlings were transferred to the same nutrient solution containing 0 or 10 μM Al for 6 h. Data are means ± SD (*n* = 3)

The rapid induction of *SlNAC* gene expression in response to Al stress led us to question whether these SlNAC TFs were early genes or late genes involved in Al tolerance in tomato. To verify this, a protein translation inhibitor, CHX, was applied before Al stress. It can be assumed that de novo protein synthesis is not required for early-gene expression activation, and thus cannot be repressed by CHX. We choose 7 among 19 Al-responsive *SlNACs* because they have higher expression levels. Intriguingly, we found that the expression of all 7 tested SlNAC TFs was substantially induced by CHX even in the absence of Al (Fig. 7), implying that there may be a transcriptional repressor which blocks the transcriptional activation of SlNAC TFs in the absence of Al, and Al stress might cause the degradation of the repressor. To exclude the possibility that the up-regulation of these 7 *SlNACs* was caused by the toxic effects of CHX, we analyzed other SlNACs expression under CHX. We found that CHX treatment could both up-regulate and down-regulate the expression of *SlNAC* genes. For example, the expression of *SlNAC056* was repressed by CHX (Fig. S3). In addition, we identified three *FRD3-like* genes in our RNA-Seq data, and found that the ability of Al to induce the expression of three *FRD3-like* genes was abolished by CHX (Fig. S4). These results suggest that these SlNAC TFs represent early genes involved in the Al stress response in tomato root apex.

## Discussion

In this present study, we systemically analyzed the NAC gene family in tomato, and identified a total of 93 *SlNAC* genes (Table S1). Numerous studies have shown that NAC TFs are widely distributed in different plant species and have potential roles in regulating plant development, growth and stress responses [15]. This family seemed to be one of the largest TFs up till now. There were 117 *NAC* genes in Arabidopsis [29], 151 in rice [30], 79 in grape [24], 180 in apple [13], 152 in maize [31], 71 in chickpea [32], 96 in cassava [26], 87 in sesame [14], 185 in Asian pears [7], and 80 in tartary buckwheat [33]. These data suggest that *NAC* genes have extensively expanded with their evolution. Therefore, phylogeny-based functional prediction is useful for functional characterization of *SlNACs*. We further divided the *SlNAC* gene family into 5 distinct subgroups based on the molecular phylogenetic analysis (Fig. 1). *SlNACs* and *AtNACs* from groups IIa, IIb, IIIa and IIIb showed that these genes were not only homologous but might even be evolved from a common ancestor. However, *NACs* from group I indicated that tomato *NAC* genes had a different ancestor from Arabidopsis (Fig. 1). Here, we provided the nomenclature of this gene family according to their chromosomal position (Table S1). As a very important vegetable and model crop for fleshy fruit development, functional genomics has become more and more popular, and studies on functional characterization of *NAC* genes in tomato have increased in recent years [34]. However, nomenclature on *NAC* genes is confusing in published studies. For example, a recent characterized tomato *SlNAP2* involved in leaf senescence and crop yield has actually been previously reported as *SlNAC35* [35, 36]. Tomato No-ripening (NOR) is NAC protein functioning as a positive regulator of fruit ripening [37]. Gao et al. (2018) identified a new NAC transcription factor named NOR-like 1, which is involved in tomato fruit ripening [38]. However, NOR-like1 is the same as SlNAC4 already characterized by Zhu et al. (2014) [39]. As summarized in Table S7, we have listed all the names of reported *SlNACs* and their corresponding names presented in this study. It might be useful for standardizing the naming of *NAC* gene in tomato for future study as well as to eliminate the naming confusion of previous studies.

It has been shown that a GWD event happened in tomato about 83–123 Myr in prior to divergence with grape [21] and this gene duplication had crucial roles in the expansion, rearrangement and functional variation of *NAC* genes [7]. In this study, there are 22 SlNAC gene pairs were found to be associated with gene duplication, including 17 GWD duplicate pairs and 5 duplicate pairs. These duplications appeared in all five groups; however, group I had only 1 pair of duplication (Fig. 4). These results suggest that both GWD and tandem duplications contributed greatly to the expansion of the *SlNAC* gene family in tomato. Furthermore, MEME showed that groups IIa, IIb, IIIa and IIIb had main similar motifs (Motif 1–Motif 7) with minor changes and exchanges. In contrast, members of group I have evolved additional motifs (Motifs 17 and 18) (Fig. 3). Gene structure analysis also illustrated no intronic regions in mostly members of group I (Fig. 2). Therefore, SlNAC genes from groups I could have functions specific to *Solanum* species and the members from this subgroup in Arabidopsis might have been lost during the evolution. Alternatively, members were independently evolved in *Solanum* species**.**

Generally, gene expression patterns are able to provide essential cues for gene function. Therefore, we determined the expression levels of the 93 *SlNAC* genes in leaf, root, flower, and fruit tissues using RNA-Seq data downloaded from the TFGD database. As shown in Fig. 5, a high and/or preferential expression of 45 *SlNACs* was detected, which displayed tissue- and development-specific expression patterns in leaf, root, flower, and breaker fruit. These genes may have important roles in growth and development of tomato and their precise functions still remains to be elucidated in further investigations. Furthermore, the expression patterns of some *SlNACs* differed in different tissues and development stages, suggesting that the SlNAC TFs may have diverse functions. In addition, we also found that 9 genes (*SlNAC25*, *SlNAC35*, *SlNAC37*, *SlNAC43*, *SlNAC47*, *SlNAC81*, *SlNAC82*, *SlNAC83*, and *SlNAC93*) were highly expressed in all the examined tissues (Fig. 5), suggesting that they may be involved in specific housekeeping activity in the growth and development of tomato. In future, more research will be needed to examine the precise functions of the *SlNAC* genes in tomato.

We identified 19 *SlNAC* genes that responded quickly to Al stress in the root apex of tomato, of which 7 were expressed in abundance in the root apex (Fig. 6). Interestingly, these 7 *SlNAC* genes belong to IIb (*SlNAC033*, *SlNAC063*, *SlNAC064*, and *SlNAC084*) and IIIb (*SlNAC019*, *SlNAC031*, and *SlNAC065*) subgroups (Fig. 1). Similarly, most of Al-responsive *NAC* genes in rice belong to NAM subgroup i.e. IIb subgroup [17]. A few members from subgroup IIb have been well characterized with respect to fruit development and responses to biotic and abiotic stresses in tomato. For example, both *SlNAC033* and *SlNAC064* have been demonstrated to be implicated in tomato fruit development possibly via regulating the expression of genes in association with ethylene biosynthesis and cell wall metabolism [38, 39]. *SlNAC063*, previously known as *JA2L*, has proven to be necessary for jasmonic acid-mediated stomatal movement during pathogenesis [40]. By contrast, much less is known of the function of NAC genes from subgroup IIIb. In *Arabidopsis*, CUC1 and CUC2 belong to this subgroup functioning redundantly to regulate shoot apical meristem formation [41]. In tomato, the *GOBLET* gene, which encodes a NAC transcription factor (assigned as SlNAC062 in this study), directs the leaflet boundaries in compound leaves of tomato [42]. The involvement of three subgroup IIIb *SlNACs* in Al stress suggests that Al might affect root apex meristem development. Alternatively, the function of NAC genes from this subgroup is not limited to development. However, their functions in Al stress tolerance need to be examined in the future.

It appears that Al-responsive *SlNAC* genes are early factors involved in Al stress responses as evidence by a protein translation inhibitor experiment. Generally, early genes are required for the transcription of secondary response or late genes. Here, we found that Al-induced expression of a tomato *FRD3-like* gene (Solyc01g087150) was almost completely blocked by CHX (Fig. S4), suggesting that this gene represents one of the late genes required for long-term responses (possibly citrate efflux) to Al stress in tomato. A similar expression regulation pattern has been reported previously in rice bean, in which VuSTOP1 expression could also be induced by CHX in the absence of Al stress, whereas Al-induced VuMATE1 expression was completely inhibited [43]. At present, the direct target gene of these SlNAC TFs remains unknown. A recent study showed that a rice bean (*Vigna umbellata*) NAC TF, VuNAR1 can activate the expressiong of cell wall related receptor kinase 1 (*WAK1*) gene that contributes to reduced pectin content in the cell wall and Al^3 +^binding capacity, thus enhancing Al tolerance. Since several lines of evidence suggest the role of NAC TFs in cell wall metabolism, it seems very likely that these SlNACs may also be involved in the Al stress response by regulating cell wall metabolism, which requires further investigation.

## Conclusions

In this study, the first integrated analysis including gene identification, structure, chromosomal location, duplications, tissue and Al response expression patterns of the *NAC* gene family in tomato was carried out. A total of 93 *SlNAC* genes were identified, which will provide essential information for the functional characterization of *SlNAC* genes in tomato. Analysis of previously published RNA-Seq data indicates that *SlNAC* genes may participate in the development of tomato. Our RNA-Seq data showed that the expression levels of 19 *SlNAC* genes were significantly changed under Al stress. These data are helpful for further investigation of *NAC* gene-mediated physiological and molecular processes involved in Al stress, and provides new insight into *NAC* gene family and a basis for further exploration on its functional mechanisms in tomato.

## Methods

### Identification of the *NAC* family genes in tomato

The Hidden Markov Model (HMM) file corresponding to the NAC domain (PF02365) was download from the Pfam protein family database (http://pfam.xfam.org/) [44]. HMMER 3.2 was used to search against the *NAC* genes from the tomato genome database from Phytozome v12.1 (https://phytozome.jgi.doe.gov/pz/portal.html) [45, 46]. All candidate genes that may contain NAC domain based on HMMER results were further examined by confirming the existence of the NAC core sequences using PFAM and the SMART program (http://smart.embl-heidelberg.de/smart/batch.pl) [47]. Length of sequences, protein molecular weights, transmembrane domains and subcellular location of identified tomato NAC proteins were obtained by using tools from ExPasy website (https://www.expasy.org/).

### Phylogenetic analysis of the NAC gene family members

The NAC domain sequences of 93 identified tomato NACs and 110 Arabidopsis NACs from PlantTFDB 4.0 4http://planttfdb.cbi.pku.edu.cn/) [48] were used to create multiple protein sequence alignments using ClustalW in MEGA 7.0 (https://www.megasoftware.net/) [49] with default parameters. The alignment results were used to construct a phylogenetic tree using the neighbor-joining method with 1000 bootstrap replicates. The phylogenetic tree was displayed with the online tools iTOL v4 (https://itol.embl.de/) [50].

### Gene structure and conserved motif analysis

The exon-intron distribution of each tomato *NAC* genes (*SlNACs*) was analyzed by comparing predicted coding sequences with their corresponding genomic sequences acording to Gene Structure Display Server 2.0 (http://gsds.cbi.pku.edu.cn/) [51]. Conserved motifs of tomato NAC protein sequences were investigated using the online software MEME5.0.4 (http://meme-suite.org/tools/meme) [52] with the following motif parameters: number of repetitions (any), maximum number of motif (20), and the optimum width of each motif, (between 6 and 100 residues).

### Chromosomal distribution and gene duplication analysis

All *SlNACs* were mapped to 12 tomato chromosomes based on physical location information from the database of tomato genome using TBtools program (https://github.com/CJ-Chen/TBtools) [53]. Multiple Collinearity Scan Toolkit (MCScanX) with the default parameters was used to analyze the tandem repeats and segmental duplication events of *SlNAC* gene family in the tomato genome (http://chibba.pgml.uga.edu/mcscan2/) [54].

### Tissue-specific expression analysis

To investigate the expression patterns of putative *SlNACs* genes in different tissues of development stages of tomato, in silico analysis of RNA-seq data [20] from Tomato Functional Genomics Database (TFGD, http://ted.bti.cornell.edu/cgi-bin/TFGD/digital/home.cgi) were carried out. Different tissues in cultivated tomato (*Solanum lycopersicum* cv. Heinz) including leaves, roots, flower buds, fully opened flowers, 1 cm, 2 cm, 3 cm, mature green, breaker, and breaker+ 10 fruits were selected as described previously [55], In the wild species (*Solanum pimpinellifolium*), ten tissues and organs, which included leaves, whole root, hypocotyl, cotyledons, flower buds, 10 days before anthesis or younger, flowers at anthesis, 10 days post anthesis (DPA) fruit, 20 DPA fruit and breaker stage ripening fruit, were selected for analysis. Digital gene expression analysis of the putative *SlNACs* was visualized using MultiExperiment Viewer (MeV) software [56].

### Plant material and growth conditions

Tomato (*Solanum lycopersicum*) cultivar Ailsa Craig (AC) (Horticulture Research International, Warwick, UK) was used in this study. Seeds were sterilized with 10% NaClO (v/v) for 15 min, then washed with sterilized water five times to remove the residual NaClO. Seeds were soaked in sterilized water overnight and then sown on agar plates containing 1/5 Hoagland nutrient solution (pH 5.5) consisting of KNO_3_ (1.0 mM), Ca (NO_3_)_2_ (1.0 mM), MgSO_4_ (0.4 mM) and (NH_4_)H_2_PO_4_ (0.2 mM), and the micronutrients NaFeEDTA (20 μM), H_3_BO_3_ (3.0 μM), MnCl_2_ (0.5 μM), CuSO_4_ (0.2 μM), ZnSO_4_ (0.4 μM) and (NH_4_)_6_Mo_7_O_24_ (1 μM), with 0.8% Agar (Sigma-Aldrich). Plates were kept in the dark at 4 °C for 2 d and then seeds were germinated in plant growth room with a daytime 16 h/24 °C and 8 h/22 °C night regime. After germination, uniform seedlings (until primary root length about 3–4 cm) were transferred to the 1/5 Hoagland nutrient solution (pH 5.0) with (NH_4_)H_2_PO_4_ concentration decreased to 10 μM either in the absence (−Al) or presence (+Al) of 5 μM Al for 6 h or 24 h in the same growth conditions. The tomato root tip (0-1 cm), basal root (1–2 cm) and leaves were collected for RNA extract and qRT-PCR analysis, root tips were used for further RNA-seq.

For the protein translation inhibitor cycloheximide (CHX), uniform AC seedlings (until primary root length about 3–4 cm) were pretreated with or without 10 μM CHX for 1 h, then transferred to 5 μM Al-containing solution or Al-free solution for 6 h (pH 5.0 with 10 μM (NH_4_)H_2_PO_4_). Root tips (0–1 cm) were collected for RNA-seq and qRT-PCR analysis.

### RNA-Seq and quantitative RT-PCR (qRT-PCR) analysis

For RNA-Seq, RNA samples were extracted from both root tips (1 cm in length) treated with or without 5 μM Al for 6 h. RNA-seq was carried out on an Illumina HiSeq Platform (http://www.bgitechsolutions.cn). Three biological replicates were performed for each treatment.

For qRT-PCR analysis, one microgram of DNA-free RNA was transcribed into first strand cDNA by PrimeScriptTM RT Master Mix (TaKaRa). The qRT-PCR was carried out with the Roche LightCyler 480 instrument using SYBR Green chemistry (Toyobo). The reaction conditions were 40 cycles at 95 °C for 15 s, 60 °C for 10 s, and 72 °C for 15 s. The primer sequences used in this study are listed in Table S8. Expression data of target genes were normalized with expression of tomato *GAPDH* [35] and *ACTIN* [57], respectively, by the ΔΔCt method. Each reaction was performed with three repeats from different biological samples.

### Statistical analysis

Student’s t-test was run in Microsoft Excel (v. 2016, Microsoft Corp., Redmond, WA, USA). Data are given as means ± standard deviation (SD) of three independent biological replicates. A *p*-value less than 0.05 (*p* < 0.05) was considered to be statistically significant.

## Supplementary information

**Additional file 1: Table S1**. Structural features of NAC family genes in tomato (*Solanum lycopersicum*). **Table S2**. Summary of RNA-Seq of tomato root tip in response to 0 or 10 μM Al for 6 h. **Table S3**. Statistics of genes and transcripts. **Table S4**. List of up-regulated differentially expressed genes (DEGs) in response to Al stress in tomato. **Table S5**. List of down-regulated differentially expressed genes (DEGs) in response to Al stress in tomato. **Table S6**. List of *SlNAC* genes whose expression was regulated by Al stress for 6 h. **Table S7**. List of published *NAC* genes in our naming system. **Table S8**. List of primers used in this study.

**Additional file 2: Figure S1**: Effects of Al treatment on root growth of tomato seedlings. **Figure S2**. Volcano plot analysis of differentially expressed genes (DEGs) in roots of tomato seedlings under Al for 6 h. **Figure S3**. Effects of a protein translation inhibitor, cycloheximide (CHX), on the expression of *SlNAC056* gene identified in our RNA-Seq data. **Figure S4**. Effects of a protein translation inhibitor, cycloheximide (CHX), on the expression of *FRD3-like* genes identified in our RNA-Seq data.

## Data Availability

The datasets supporting the conclusions of this article are included within the article and its additional files. RNA-Seq data is available as accession number SRP227100–1005 in the NCBI SRA database (https://www.ncbi.nlm.nih.gov/sra/SRP227103). Tomato (*Solanum lycopersicum*) cultivar Ailsa Craig (AC) used in this study is deposited in our Lab.

## Abbreviations

TF: Transcription factor
STOP1: Sensitive to proton rhizotoxicity 1
ALMT1: Al-activated malate transporter 1
CAMTA2: CALMODULIN-BINDING TRANSCRIPTION ACTIVATOR2
ART1: Al resistance transcription factor 1
NAM: No apical meristem
ATAF 1/2: Arabidopsis transcription activator factor 1/2
CUC2: Cup shaped cotyledon
WAK1: Wall-associated protein kinases
SOG1: SUPPRESSOR OF GAMMA RESPONSE1
HMM: Hidden Markov model
MCScanX: Multiple Collinearity Scan Toolkit
DPA: Days post anthesis
MeV: MultiExperiment Viewer
CHX: Cycloheximide
