# Supplementary figures and images for: Genome-wide identification and expression analysis of the NAC transcription factor family in tomato (Solanum lycopersicum) during aluminum stress
Source: BMC Genomics. 2020 Apr 7;21:288. doi: 10.1186/s12864-020-6689-7 (PMC7140551; doi:10.1186/s12864-020-6689-7)

Fig S1


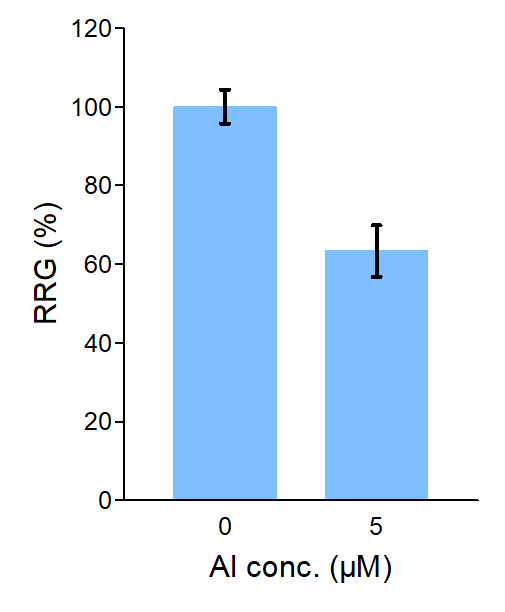


Fig. S2


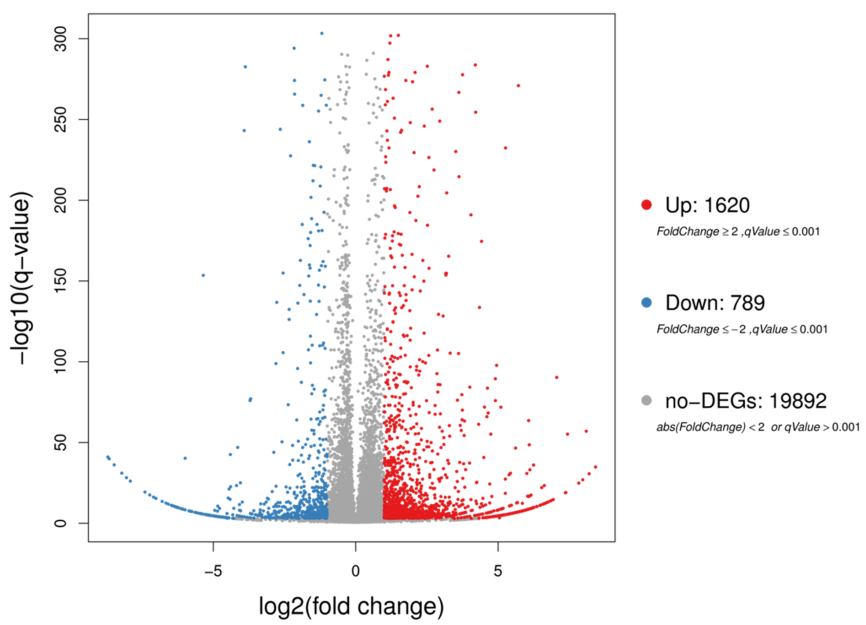


Fig. S3


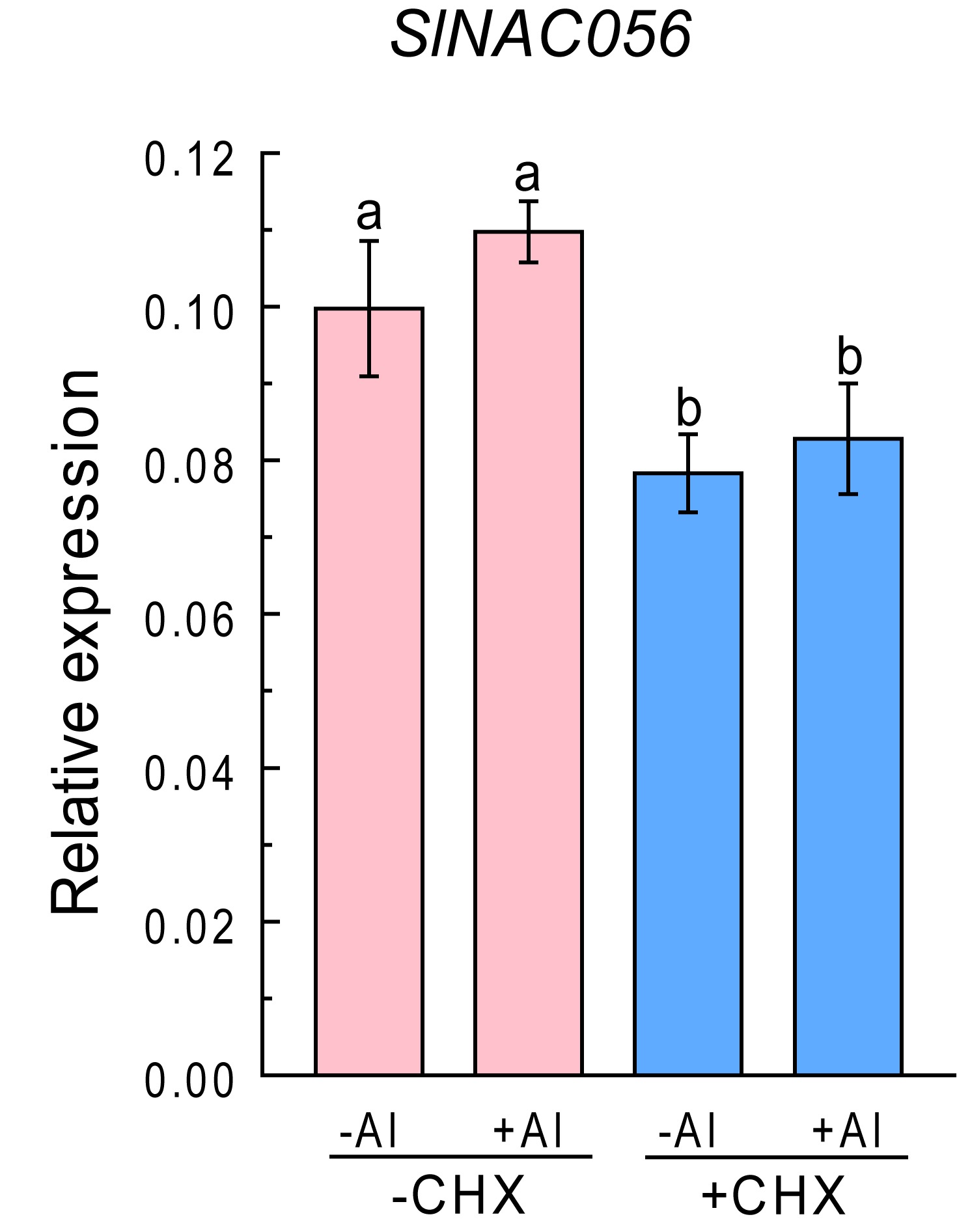


Fig. S4


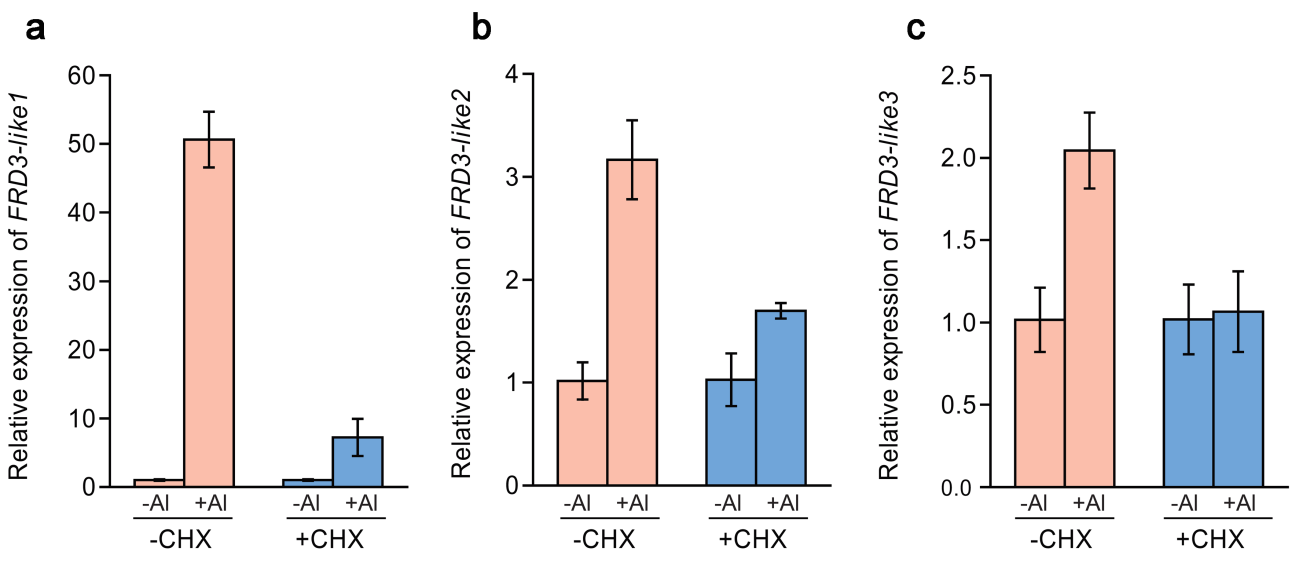

Supplement: Supplementary file 2 — Additional file 2: Figure S1: Effects of Al treatment on root growth of tomato seedlings. Figure S2. Volcano plot analysis of differentially expressed genes (DEGs) in roots of tomato seedlings under Al for 6 h. Figure S3. Effects of a protein translation inhibitor, cycloheximide (CHX), on the expression of SlNAC056 gene identified in our RNA-Seq data. Figure S4. Effects of a protein translation inhibitor, cycloheximide (CHX), on the expression of FRD3-like genes identified in our RNA-Seq data. [file 12864_2020_6689_MOESM2_ESM.doc]
